# Proteomic analysis of the effects of exogenous calcium on hypoxic-responsive proteins in cucumber roots

**DOI:** 10.1186/1477-5956-10-42

**Published:** 2012-07-12

**Authors:** Lizhong He, Xiaomin Lu, Jing Tian, Yanjuan Yang, Bin Li, Jing Li, Shirong Guo

**Affiliations:** 1 College of Horticulture, Nanjing Agriculture University/Key Laboratory of Southern Vegetable Crop Genetic Improvement, Ministry of Agriculture, Nanjing, 210095, P. R. China; 2 Anhui Science and Technology University, Fengyang, 233100, An Hui, P. R. China

**Keywords:** Cucumber, Calcium, Hypoxic stress, Proteomics

## Abstract

**Background:**

Hypoxia acts as a plant stress factor, particularly in cucumbers plants under hydroponic culture. Calcium is involved in stress signal transmission and in the growth of plants. To determine the effect of exogenous calcium on hypoxic-responsive proteins in cucumber (*Cucumis sativus* L. cv. Jinchun No.2) roots, proteomic analysis was performed using two-dimensional electrophoresis (2-DE) and mass spectrometry.

**Results:**

Cucumber roots were used to analyze the influence of hypoxia on plants. The expressions of 38 protein spots corresponding to enzymes were shown to change in response to hypoxia. Of these, 30 spots were identified by matrix-assisted laser desorption/ionization-time of flight mass spectrometry (MALDI-TOF/TOF MS analysis). The proteins were categorized according to functional groups, including glycolysis, the tricarboxylic acid (TCA) cycle, fermentative metabolism, nitrogen metabolism, energy metabolism, protein synthesis and defense against stress. Exogenous calcium appeared to alleviate hypoxic stress via these metabolic and physiological systems. Western blotting was used to analyze the accumulation of alcohol dehydrogenase (ADH) and pyruvate decarboxylase (PDC); calcium further increased the expression of ADH and PDC under hypoxia. In addition, semi-quantitative reverse transcription-polymerase chain reaction (RT-PCR) was used to assess the transcript levels of differentially expressed proteins.

**Conclusions:**

Exogenous calcium enhanced the expression of enzymes involved in glycolysis, the TCA cycle, fermentative metabolism, nitrogen metabolism, and reactive oxygen species (ROS) defense in plants under hypoxia. Calcium appears to induce hypoxic tolerance of cucumber seedlings. These phenomena have prompted us to further investigate the mechanisms by which cucumbers respond to exogenous calcium under hypoxia.

## Background

Cultivated plants that produce vegetables, crops and fruits are frequently subjected to submerged conditions (so-called hypoxia) caused by flooding [1], waterlogging [2], irrigation or hydroponic culture [3]. Plants subjected to hypoxia undergo dramatic metabolic changes and induce defensive mechanisms to cope with the potential damage caused. Hypoxia induces enhanced aerenchyma formation, stem elongation, gas film around submerged-leaves [4] and shoot biomass [5]. The first process to be influenced by the metabolic change induced by a shortage of oxygen is respiration. Hypoxic stress interferes with the electron transport system causing a lack of suitable electron acceptors, which appears to be linked to the saturated conditions of a redox state, accumulation of NAD(P)H and suppressed synthesis of ATP [6]. Energy from respiratory metabolism is necessary for the growth and yield of plants. Cucumber plants are sensitive to hypoxia, which frequently causes large reductions in yield [7].

Exogenous calcium can improve the suppression of growth/development of plants and help to maintain cell function by relieving gene repression caused during salt stress [8], anoxia [9], and chilling [10]. The involvement of calcium in oxygen debt responses is also observed in many plants. For example, the oxygen debt (anoxia) in cells of maize, rice and wheat plants causes a rise in cytoplasmic Ca^2+^ concentration [11,12]. In addition, elevated calcium levels significantly influence metabolic fluxes and substrate oxidation under hypoxic condition [13]. According to our previous research [14], exogenous calcium enhances the biomass and soluble protein content of cucumber seedlings under hypoxia (Additional file 1: Table S1). Thus, calcium appears to act as a signaling component during anoxia signal transduction in plants. The alteration in Ca^2+^ concentration seems to decrease cytosolic pH, which probably represents a major signal in cells under suspension culture and in intact seedlings [15]. However, the mechanism of calcium’s involvement in resistance to hypoxia remains unclear.

Proteomic analysis, commonly using mass spectrometry (MS), is a powerful technique that facilitates the visualization and comparison of complex mixtures of proteins. Proteomic analysis provides a large amount of information on individual proteins involved in specific biological responses. Recently, there have been many proteomic studies of cucumber plants [7,16-22]; however, none of them investigated the effects of exogenous calcium on the proteome of cucumber seedlings under root-room hypoxia stress. The purpose of the present study is to use proteomics to clarify the relationship between calcium and proteins in cucumber plants under hypoxic stress. We found that exogenous calcium could enhance both responsive metabolism and fermentative metabolism of cucumber seedlings, improving their tolerance to hypoxia. The results should provide a basis for future studies at both the physiological and molecular levels.

## Results and discussion

### Identification and functional classification of proteins by MALDI-TOF/TOF

**Figure 1:**
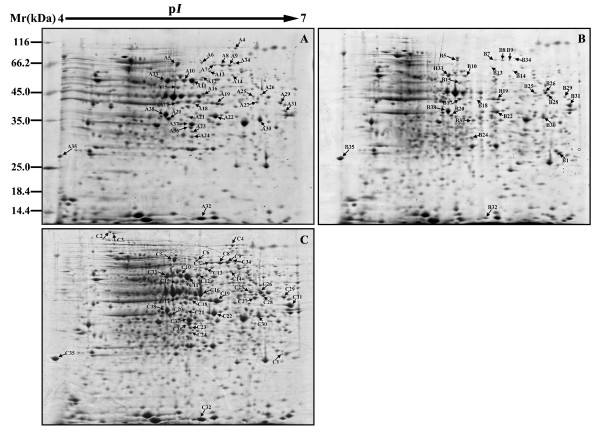
**Two-dimensional electrophoresis gel of separated proteins at three treatments. **The 2-DE protein profiles of cucumber seedling roots under normoxic (**A**), hypoxia (**B**) and hypoxia + CaCl_2 _(**C**). The expression of numbered proteins was attended significantly and these proteins were identified by MALDI-TOF/TOF MA (see Tables 1 and 2).

To examine the effect of exogenous calcium on the proteome of cucumber seedlings under hypoxic stress in water culture, 2-DE analysis of total proteins in the roots was performed. Root proteins were extracted from cucumber seedlings exposed to normoxic, hypoxic and hypoxic + 4 mM CaCl_2_ (hypoxic + Ca^2+^) conditions. Proteins purified from roots were separated by 2-DE and analyzed by Imagemaster™ 2D Platinum software; the p*I* value and molecular masses of these protein spots ranged from 4 to 7 and from 14.4 to 116.0 kDa, respectively. The characteristics of proteins in representative gels are shown in Figure 1 and described below.

Approximately 500 spots were detected in Coomassie blue (CBB)-stained gels. Thirty-eight of these spots showed significant changes in relative volume (>1.5-fold) and were excised from the gels for MALDI-TOF/TOF MS analyses.

Thirty spots were identified using the NCBI viridiplantae database (V.2010.12.10, 184045 sequences) and the NCBI EST viridiplantae database (V.2010.12.10, 1847412 sequences), giving an identification success rate approximately 79%. The results are summarized in Table 1. Four proteins (spots 2, 11, 12 and 36) expressed under normoxic and hypoxia + CaCl_2_ conditions did not appear to be expressed in hypoxia-treated plants. Spot 28 was present in plants under hypoxia and hypoxia + CaCl_2_, but was not present in plants under normoxic condition. Sixteen proteins (spots 4, 5, 8, 10, 13, 15, 16, 17, 18, 20, 22, 25, 27, 30, 32 and 37) were downregulated in hypoxia-treated plants, but upregulated in hypoxia + Ca^2+^ treated plants. Four proteins (spots 24, 33, 34 and 38) were downregulated under hypoxia and hypoxia + CaCl_2_ condition. The expressions of two proteins (spots 14 and 19) under hypoxia + CaCl_2_ conditions were significantly greater than those under normoxic and hypoxic conditions. Two proteins (spot 26 and 31) were upregulated under hypoxic conditions and further upregulated under hypoxia + CaCl_2_ conditions. Spot 1 was significantly accumulated under hypoxic and normoxic conditions.

**Table 1:** Differentially expression proteins identified by MALDI-TOF/TOF MS

^**a**^**Spot No.**	**Protein name**	**Groups**	**Accession No.**	**Plant species**	^**b**^**Mr/P***I*	**Score**	^**c**^**PM**	^**e**^**Cov (%)**	^**f**^**protein expression (%Vol)**
									**A B C**
1	JGCCJG2048B02.b Jatropha curcas L. germinating seeds (mixed stages) Jatropha curcas cDNA clone	EST sequence	gi|302362663	*Jatropha curcas*	26.6/7.26	135	4	6.27	
2	Unnamed protein product	Other	gi|9759529	*Arabidopsis thaliana*	132.67/5.4	78	23	16.90	
4	Aconitate hydratase, cytoplasmic	Citric acid cycle	gi|1351856	*Cucurbita maxima (winter squash)*	98.57/5.74	184	16	19.04	
5	V-type proton ATPase catalytic subunit A	Energy metabolism	gi|401322	*Gossypium hirsutum (upland cotton)*	68.76/5.36	270	19	33.87	
8	phosphoglycerate mutase	Glycolysis	gi|32400802	*Triticum aestivum (bread wheat)*	29.62/5.43	169	8	30.43	
10	Enolase	Glycolysis	gi|14423688	*Hevea brasiliensis*	48.0/5.57	180	8	21.75	
11	Enolase	Glycolysis	gi|1169534	*Ricinus communis (castor bean)*	48.1/5.56	266	10	25.84	
12	starch synthase III	Energy metabolism	gi|9502143	*Triticum aestivum (bread wheat)*	184.0/4.94	68	22	12.10	
13	TransId-212581 CACATN1 Coffea arabica cDNA clone	EST sequence	gi|257024642	*Coffea arabica*	32.1/10.27	87	12	12.31	
14	CBOZ5962.b1 CBOZ Coccomyxa sp. C-169 8 kb Coccomyxa sp. C-169 cDNA clone	EST sequence	gi|282500599	*Coccomyxa subellipsoidea C-169*	25.4/10.03	85	12	16.27	
15	CLS_cLiFproots_25a3_1_h11cLibkit5LD_D06 CLS_cLiFproots_plant Festuca arundinacea cDNA clone	EST sequence	gi|257183562	*Festuca arundinacea*	39.4/9.76	88	13	10.27	
16	cofactor-independent phosphoglyceromutase	Glycolysis	gi|6706331	*Apium graveolens*	61.1/5.26	112	7	12.27	
17	putative protein phosphatase 2 C	Protein synthesis	gi|50725575	*Oryza sativa Japonica Group*	34.6/4.88	66	4	14.15	
18	galactokinase	Glycolysis	gi|53747925	*Pisum sativum*	55.2/5.4	78	3	5.84	
19	Glutamine synthetase cytosolic isozyme	Nitrogen metabolism	gi|12643762	*Lotus japonicus*	39.3/5.49	95	7	24.44	
20	pyruvate dehydrogenase2	Citric acid cycle	gi|162464059	*Zea mays*	40.1/5.54	184	5	12.06	
22	Malate dehydrogenase, cytoplasmic	Citric acid cycle	gi|11133373	*Medicago sativa*	35.9/6.39	624	11	48.49	
24	CLS_cLiFproots_52a4_1_b18cLibkit5LD_ A09 CLS_cLiFproots_plant Festuca arundinacea cDNA clone	EST sequence	gi|257180604	*Festuca arundinacea*	41.9/10.49	100	14	11.14	
25	Os03g0851100	Other	gi|115456623	*Oryza sativa Japonica Group*	48.6/6.04	77	7	15.45	
26	alcohol dehydrogenase	Fermentative metabolism	gi|52851054	*Populus tremula*	33.1/6.08	173	7	24.44	
27	putative pyruvate dehydrogenase e1 alpha subunit	Citric acid cycle	gi|13430788	*Arabidopsis thaliana*	43.5/7.15	178	13	34.20	
28	methionyl-tRNA synthetase	Protein synthesis	gi|4091008	*Oryza sativa (rice)*	90.9/6.55	78	15	18.66	
30	fructose-bisphosphate aldolase, class I	Glycolysis	gi|15227981	*Arabidopsis thaliana*	38.7/7.01	282	8	27.37	
31	peroxidase	Defense against stress	gi|167531	*Cucumis sativus (cucumber)*	32.7/6	96	2	5.78	
32	Os02g0121900	Other	gi|115443885	*Oryza sativa Japonica Group*	70.3/9.39	73	14	28.69	
33	F1-ATP synthase, beta subunit	Energy metabolism	gi|4388533	*Sorghum bicolor*	49.2/5.25	898	16	45.81	
34	Os06g0597200	Other	gi|115468776	*Oryza sativa Japonica Group*	40.1/5.32	78	6	12.66	
36	putative fructokinase	Glycolysis	gi|14423528	*Arabidopsis thaliana*	35.4/5.3	221	4	13.54	
37	FS080420 library SmFL Solanum melongena cDNA clone	EST sequence	gi|261665622	*Solanum melongena*	21.2/10.18	88	12	19.02	
38	GSTSUB_UP_031_F12_01SEP2004_086 GSTSUB Artemisia annua cDNA, mRNA sequence	EST sequence	gi|283968778	*Artemisia annua*	23.1/10.1	106	11	14.81	

^a^Spot numbers are given in Figure 1.

^b^Theoretical molecular mass (Mr) and isoelectric point (pI) of the identified proteins.

^e^The percent coverage of peptides.

^f^he relative levels of protein expression. A: normoxic; B: hypoxia; C hypoxia + CaCl_2_.

Eight identified spots (spots 1, 2, 13, 14, 15, 24, 37 and 38) were annotated either as unnamed proteins or ESTs in the databases, and three spots (spots 25, 32, 34) were described in The Rice Annotation project Database (RAP-DB). We searched for their homologs using BLAST http://www.ncbi.nih.gov/BLAST/ and their protein or nucleotide sequences as queries. The six proteins showing the highest similarity are listed in Table 2. These similar proteins showed more than 85% positives at the amino acid level, indicating that they might have similar functions. The remaining 19 identified proteins were involved in various biological processes and could be classified into three groups [23,24]. The first group consists of proteins involved carbon metabolism, nitrogen metabolism, and energy metabolism. The second group consists of regulatory proteins involved in translation and synthesis. The third group consists of proteins participating the stress response. 

**Table 2:** Homologs of unknown proteins

**Spot No.**	**Accession No.**^**a**^	**Homologue**				
		**NCBI accession No.**^**b**^	**Protein Name**	**Plant species**	**Ident**^**c**^	**Pos**^**d**^
B1	gi|302362663	CAI83772.1	glyceraldehyde-3-phosphate-dehydrogenase	*Lupinus albus*	93%	97%
2	gi|9759529	NP_200612.2	FIP1 [V]-like protein	*Arabidopsis thaliana*	99%	99%
13	gi|257024642	ACD03224.1	xyloglucan endotransglucosylase	*Actinidia deliciosa*	77%	91%
25	gi|115456623	AAG32661.1	translational elongation factor EF-TuM	*Zea mays*	89%	94%
32	gi|115443885	XP_003573599.1	pentatricopeptide repeat-containing protein At1g02060	*Brachypodium distachyon*	80%	90%
34	gi|115468776	BAD33043.1	putative protein phosphatase 2 C	*Oryza sativa Japonica Group*	100%	100%

^a^The gi number of the unknown proteins. ^b^The accession number of homologues. ^c^Identities. ^d^Positives.

### Structural proteins and enzymatic proteins involved in energy metabolism

Most of the identified proteins were structural proteins (non-enzymatic proteins) and enzymes involved in energy metabolism. These enzymes seem to have particularly important roles in cucumber plants under hypoxic conditions. The expressions of cytoplasmic aconitate hydratase (spot 4), pyruvate dehydrogenase 2 (spot 20), cytoplasmic malate dehydrogenase (spot 22) and pyruvate dehydrogenase e1 alpha subunit (spot 27) from the TCA cycle were downregulated under hypoxic conditions, but upregulated under hypoxia + CaCl_2_ conditions. The TCA cycle is a key component of the metabolic pathway by which all aerobic organisms generate energy by oxidization of pyruvate into carbon dioxide and water. Pyruvate dehydrogenase 2 (PDH2), a pyruvate dehydrogenase E1 beta isoform, and pyruvate dehydrogenase e1 alpha subunit are involved in the formation of cellular energy through the TCA cycle and in the synthesis of acetylcholine (acety1-CoA). Acetyl-CoA may then be used in the TCA cycle to carry out cellular respiration; thus, pyruvate dehydrogenase links the glycolytic pathway to the TCA cycle and releases energy via NADH. Calcium activates pyruvate dehydrogenase, isocitrate dehydrogenase (IDH) and α-ketoglutarate dehydrogenase [25]. Aconitate hydratase (aconitase) catalyses the stereospecific isomerization of citrate to isocitrate via cis-aconitate in the TCA cycle [26,27]. Malate dehydrogenases (MDH), which is essential to the TCA cycle, catalyses the conversion of oxaloacetate to malate [28]. Increased malate levels in plants contributes to plant acid resistance and aluminum toxicity tolerance [29]. In apples and tomatoes, significant accumulation of malate dehydrogenases gene transcripts is related to plant and cell growth, as well as to tolerance of salt stress [30]. The activities of enzymes such as succinate dehydrogenase (SDH), isocitrate dehydrogenase (IDH) and malate dehydrogenase (MDH) in the TCA cycle are different between the hypoxia and hypoxia + CaCl_2_ conditions, where exogenous calcium promotes the actives of SDH and IDH in cucumber [31]. Moreover, calcium acts to maintain higher activities of MDH and SDH and a certain level of aerobic respiration in pepper [32]. Thus, exogenous calcium seems to induce tolerance to hypoxia in cucumber plants through the activation of the enzymes involved in the TCA cycle.

Cytosolic enolase is expressed in many plant species in response to various environmental stresses, such as salt stress [33], cold [34,35] and drought [36]. However, the level of protein expression appears not to be correlated with the enzyme’s activity. Although enolase activity was increased by the stresses, the level of expression of the enolase protein showed no fluctuation [37] or was even observed to significantly decrease [33]. In the present study, this enolase (spots 10 and 11) appeared to be downregulated under hypoxia compared to hypoxia + CaCl_2_ and control. These results imply that enolase activity is regulated at the posttranscriptional level under anaerobic conditions and its relative amount is increased by exogenous calcium during hypoxia.

The expression of phosphoglycerate mutase (PGAM) (spot 8) and cofactor-independent phosphoglyceromutase (iPGAM) (spot 16) markedly decreased under hypoxic stress, but increased on the addition of exogenous calcium. PGAM is a key enzyme in glycolysis, catalyzing the interconversion of the phosphate group from C-3 to C-2, which results in the conversion of 3-phosphoglycerate (3PGA) to 2-phosphoglycerate (2PGA). PGAMs are divided into two evolutionarily unrelated groups based on whether they require 2, 3-biphosphoglycerate as a cofactor: cofactor dependent PGAMs (dPGAMs) and cofactor-independent PGAM (iPGAMs). The iPGAMs are commonly present in higher plants, some invertebrates, fungi, and bacteria [38]. PGAMs are important to stomatal movement, vegetative biomass production, and reproduction in *Arabidopsis*[39]. Transgenic potato plants with reduced iPGAM enzyme activity showed reduced growth because of a reduced photosynthetic rate [40]. These phenomena suggest that the conversion of 3PGA to 2PGA may be inhibited under hypoxia, and that exogenous calcium may increase the abundance of the proteins.

Fructose-bisphosphate aldolase (FBP aldolase, spot 30) is also an essential enzyme involved in glycolysis. It catalyzes a reversible cleavage reaction of fructose-1, 6-bisphosphate (F-1, 6-BP) into two trioses: glyceraldehydes-3-phosphate and dihydroxyacetone phosphate (DHAP) [41]. Increased FBP aldolase activity stimulates the glycolytic pathway and plays an important role in gibberellin A (GA)-induced growth of rice roots [42] and in signal transduction [43]. In the present study, downregulation of FBP aldolase under the hypoxia altered the levels of glycolysis and inhibited the growth of cucumber roots. Exogenous calcium significantly elevated the quantity of the FBP aldolase, which may help alleviate the effects of hypoxic stress. This result is consistent with the expression profile of this protein in cucumber roots under salt stress [20].

The glycolytic pathway is the major source of energy when oxygen availability decreases below the level at which oxygen becomes limiting for oxidative phosphorylation [44]. Pyruvate produce from glycolysis is consumed by fermentative metabolism, which involves pyruvate decarboxylase (PDC) and alcohol dehydrogenase (ADH, spot 26). ADH catalyzes the reduction of pyruvate to ethanol and results in continuous NAD^+^ regeneration. ADH is considered essential for survival of plants during anaerobic conditions [45]. Ruthenium red, an organelle calcium channel blocker, dramatically reduced anoxia-induced ADH activity [46] and gene expression [47]. As expected, the quantity of ADH was increased under hypoxia and increased further under hypoxia + CaCl_2_.

Spot 12, spot 18 and spot 36 were identified as starch synthase III (SSIII), galactokinase and fructokinase, respectively. SS is involved in the elongation of the linear chains of starch [48]. SSIII specifically catalyzes the formation of chains with a degree of polymerization (DP) of 12 to 25. Other SS isoforms cannot fulfill this function [49]. Galactokinase is involved in the conversion of stachyose to sucrose in the cucumber peduncle [50]. Fructokinase specifically catalyzes the transfer of a phosphate group from ATP (the substrate) to fructose as the initial step in its utilization. Recent studies have suggested that sucrose and hexoses (mainly glucose and fructose) can act as sensing-molecules to elicit sugar responses in both source and sink organs when plants are under abiotic stress [51], and can control distinct aspects of plants’ development [52]. In the present study, these enzymes were significantly downregulated under hypoxia, but upregulated under hypoxia + CaCl_2_. A decrease of starch or carbon metabolic activity was also observed in other studies using various plant species under hypoxia [44,53]. These observations provide a convenient explanation of the adaptive response of plants to hypoxia, namely that plants limit their energy consumption by suppressing the synthesis of storage substances, such as starch and protein [54,55]. Calcium seems to enhance carbohydrate metabolism and induces sugar signaling to enhance tolerance of cucumber plants subjected to hypoxic stress.

According to recent studies using transgenic plants, overexpression or altered expression of glutamine synthetase (GS) promotes the development of plants [56] such as wheat [57] and *Lotus corniculatus*[58]. The expression of this protein (GS, spot 19) decreased under hypoxia, but was significantly enhanced under hypoxia + CaCl_2_. Thus, calcium appears to regulate nitrogen (N) metabolism through GS to relieve O_2_-deficient conditions in cucumber plants subjected to hypoxia.

ATP synthases (ATPases) are membrane-bound enzyme complexes/ion transporters that combine ATP synthesis and/or hydrolysis with the transport of protons through the membrane [20], playing a key role in biological energy metabolism. ATPases differ in respect to function (ATP synthesis and/or hydrolysis), structure (F-, V- and A-ATPases contain rotary motors) and in the type of ions they transport [59,60]. Two ATPases, i.e. V-type proton ATPase catalytic subunit A (spot 5) and F1-ATP synthase, beta subunit (spot 33) were remarkably decreased under hypoxia. Under hypoxia + CaCl_2_, the level of the former (spot 5) was restored and the latter (spot 33) showed a tendency to be somewhat restored. These restored levels did not reach the level of the control (normoxic conditions). V-type proton ATPases generate a proton electrochemical gradient, which is the driving force utilized by the tonoplast Na^+^/H^+^ antiporter, to compartmentalize Na^+^ into the vacuole [61]. F1-ATPases in mitochondria, chloroplasts and bacterial plasma membranes are the prime producers of ATP, using the proton gradient generated by oxidative phosphorylation (mitochondria) or photosynthesis (chloroplasts). Mitochondrial Ca^2+^ accumulation triggers activation of mitochondrial metabolism, which increases ATP synthesis in mitochondria and ATP levels in cytosol [62]. This phenomenon suggests that hypoxia dramatically inhibits energy metabolism in cucumber plants, and in the case of these two ATPases, calcium cannot completely restore them to normoxic levels.

Calcium is an essential element for cell growth and plays a role as a second messenger in signal transduction pathways [63]. Therefore, it is not surprising that calcium is implicated in plant metabolism regulation signaling, particularly in association with oxygen deprivation [64]. According to Gao et al., exogenous calcium induces the promotion of physiologically active factors and matters in muskmelon plants, as compared to the factors and matters observed in plants under hypoxic stress after 6 days [65]. CaCl_2_ pretreatment increased the accumulation of amino acids in rice roots under anaerobic stress, possibly via a Ca-Camodulin complex involved in the transduction of an anaerobic signal that inhibits proteolysis and solute release [9]. In addition, downregulation of a suite of energy metabolic pathways, and therefore, oxygen-consumption, is a class of plant hypoxic responses [66]. In the present study, enzymes of carbon and nitrogen metabolism in the cytosol, mitochondria and chloroplasts were significantly induced by exogenous calcium. Thus, calcium enhances the tolerance of cucumber plants under hypoxia by regulating metabolic systems in the glycolytic pathway and the TCA cycle, and the activity of enzymes, such as ADH and GS. Although exogenous calcium had only a slight effect on ATPases, this effect seems to be part of the global effect of calcium on metabolism in cucumber plants.

### Regulatory proteins

Plant growth and productivity is suppressed by hypoxic stress or flooding [67]. As the cell metabolism adapts to hypoxia, increased protein degradation might control the levels of one or more regulators/enzymes [68]. Protein phosphatase 2C (PP2Cs, spot 17) decreased under hypoxia and increased under hypoxia + CaCl_2_. This enzyme is a negative regulator of stress signaling in plants and mammals [69] and acts predominantly through the signaling pathway of the stress hormone, abscisic acid (ABA) [70]. Thus, the increase in the amount of the enzyme under hypoxia + CaCl_2_ may imply that the exogenous calcium influences ABA signaling to relieve hypoxic stress. Spot 28 was identified as methionyl-tRNA synthetase (MetRS). This enzyme is a multi-domain protein that specifically binds tRNA^Met^ and catalyzes the synthesis of methionyl-tRNA^Met^[71], giving it a vital role in protein biosynthesis. The MetRS gene has been described in the mitochondria and chloroplasts of *Arabidopsis thaliana*[72] and has been linked with plant cell anti-oxidant defense during oxidative stress [73]. Although MetRS was not detected under normoxic conditions, it was induced under hypoxia and further increased under hypoxia + CaCl_2_. This result suggests that the expression of MerRS under hypoxia represents a stress response of the cucumber seeding and calcium enhanced this response under hypoxic stress.

### Proteins related to the stress response

Excessive generation of reactive oxygen species (ROS) or oxidative stress is an integral part of many stress situations, including hypoxia [74]. Higher plants have active oxygen-scavenging systems, consisting of multiple defense enzymes that can modulate the steady-state level of ROS [75]. Peroxidase (POD, spot 31), a ubiquitous enzyme [76] present in plants, microbes, fungi and vertebrates. This enzyme acts as a biological catalyst to scavenge H_2_O_2_[77]. The activities and gene expressions of superoxide dismutase (SOD) and ascorbate peroxidase (APX) were increased in pigeon pea [78], mung bean [74] and cucumber [79,80] under waterlogged conditions. These phenomena were consistent with the changes in the expression of POD in the present study. In addition, the expression of POD was further increased by treatment with calcium. Thus, exogenous calcium can enhance the activities of ROS-scavenging enzymes to defend against the damage caused by ROS, which would suppress the effects of hypoxic stress.

### Validation of differentially expressed proteins by western blotting

**Figure 2:**
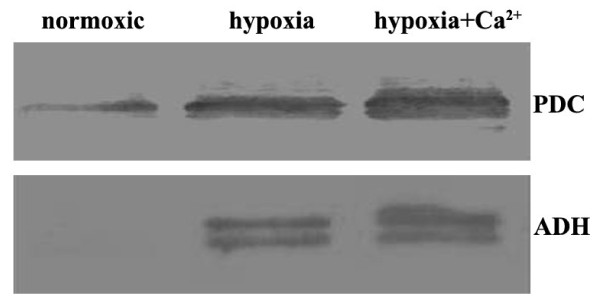
**Western blot analysis of PDC and ADH expression level under three treatments**.

To maintain ATP levels in plants under hypoxia, the plants seem to continuously regenerate of NAD^+^ in the cytosol (glycolysis) and mitochondria (TCA cycle). Ethanol formed by alcohol fermentation diffuses rapidly out of the cells, inducing a considerable loss of carbon during hypoxia. In this regard, pyruvate decarboxylase (PDC) and alcohol dehydrogenase (ADH) are considered as important plant proteins for coping with hypoxia-induced cellular damage [81]. According to Kang et al. [82], 24-epibrassinolide (EBR) further enhances ADH activity in hypoxic cucumber roots. Therefore, PDC and ADH were analyzed by western blotting to verify the proteomic data. As shown in Figure 2, the expression of PDC and ADH seems to change among plants grown under the three culture conditions. The PDC protein levels were upregulated under hypoxia, as compared to normoxic (control) conditions, and was further upregulated under hypoxia + CaCl_2_. Under hypoxia and hypoxia + CaCl_2_, ADH showed a similar tendency that of PDC, although the level of ADH under normoxic conditions was very low. The western blot results correspond well with the proteomic results and were consistent with previous research. 

### Transcript accumulation patterns for 12 candidate proteins

**Figure 3:**
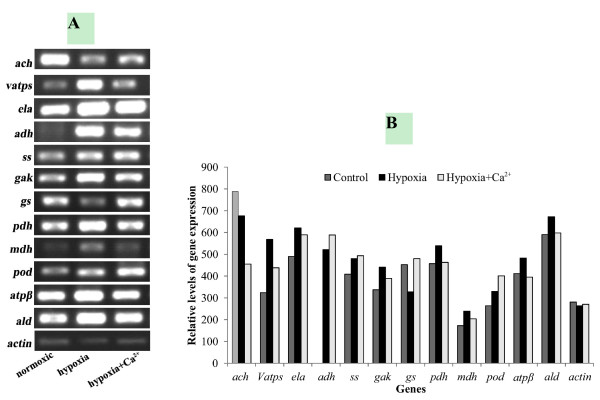
**RT-PCR analysis of transcript levels of differentially expressed proteins under three treatments**. ach: aconitate hydratase; vatps: V-type proton ATPase; ela: enolase; adh: alcohol dehydrogenase; ss: starch synthase; gak: galactokinase; gs: glutamine synthetase; pdh: pyruvate dehydrogenase; mdh: malate dehydrogenase; pod: peroxidase; atpβ: F1-ATP synthase, beta subunit; ald: aldolase. Transcript levels were measured three days after the treatments (**A**), and the relative abundance ratio of the genes was analyzed (**B**). A single concentration of cDNA was also used for amplification with ACTIN (AF171095, actin) primers. ACTIN was used as the internal standard to determine the extent of cDNA amplification.

RT-PCR was used to analyze the changes in gene expression at the mRNA level of 12 identified proteins involved in glycolysis, the TCA cycle, energy metabolism, nitrogen metabolism, fermentative metabolism and defense against stress (Figure 3,A and B). Different peptide sequences obtained from protein spots were used to design primers to compare mRNA accumulation under control, hypoxia and hypoxia + CaCl_2_ conditions, 3 days after treatment. As shown in Figure 3A and B, the mRNA levels of seven transcripts (*vatps*, *ela*, *adh*, *gas*, *pdh*, *mdh*, *atpβ*, and *ald*) increased under hypoxia and decreased under hypoxia + CaCl_2_; *adh* expression was not detected under normoxic conditions. The gene expressions of *ss* and *pod* increased under hypoxic stress and further increased under hypoxia + CaCl_2_. The *ach* gene showed an opposite tendency. Thus, the mRNA levels did not correspond with the protein levels. This is not surprising, because the final amount and activity of a protein represents an accumulation regulatory events at their transcriptional, post-transcriptional, translational, and post-translational levels [83]. Therefore, the validity of estimating gene expression levels using protein expression data requires further study. 

## Conclusions

Proteomic analysis is an effective means for clarifying protein expression patterns and permits the identification of candidate proteins. In the present study, calcium was demonstrated to be involved in the short-term hypoxic tolerance of cucumber plants. Exogenous calcium enhanced the expression of proteins involved in glycolysis, the TCA cycle, nitrogen metabolism, protein synthesis, fermentative metabolism and ROS defense. This phenomenon suggests that exogenous calcium could induce hypoxia tolerance by improving enzyme activity in systems related to respiratory metabolism and stress defense in cucumber plants. However, western blotting and RT-PCR analyses showed different results for the candidate proteins. In general, exogenous calcium improves the hypoxia tolerance of plants via multiple systems that are regulated by multiple genes relating to various metabolic and signaling pathways. The present study provides evidence of the mitigating effect of exogenous calcium on the growth and metabolic activities of cucumber plants restrained under hypoxia. Further proteomic studies in this area are clearly warranted and are ongoing.

## Methods

### Plant materials and growth conditions

Cucumber (*Cucumis sativus* L. cv. Jinchun No.2, hypoxia sensitive [84]) seeds were sterilized with 0.5% (W/V) sodium hypochlorite solution for 10 min and then washed thoroughly with deionized water. The washed seeds were sown on two layers of wet filter paper and incubated in the dark at 28°C for 24 h. The germinated seedlings were transplanted to plastic trays (41 × 41 × 5 cm) containing quartz sand and grown at 25–30°C (day) and 15–18°C (night), with 60–75% relative humidity (RH), in a greenhouse of Nanjing Agriculture University in 2010. The seedlings were supplied with 1/2-strength Hoagland’s nutrient solution (pH 6.5 ± 0.1, EC 2.0–2.2 dS m^−1^). At the 2nd leaf development stage, relatively uniform seedlings were transferred to tanks containing full strength Hoagland’s nutrient solution. The solution was renewed every 3 days. The solution in the tanks was kept at 20–25°C and aerated with an air pump to keep the dissolved oxygen (DO) level at 8.0 ± 0.2 mg L^−1^ (the optimum DO level for cucumber). At the 3rd leaf development stage, seedlings were subjected to one of three treatments. (1) Control: 1/2 Hoagland’s solution (containing 2 mM Ca^2+^) with DO of 8.0 ± 0.2 mg L^−1^. (2) Hypoxia treatment: 1/2 Hoagland’s solution (containing 2 mM Ca^2+^) with DO of 1.0 ± 0.1 mg L^−1^, which was prepared by pumping N2-gas into the nutrient solutions as the hypoxic treatment. The oxygen concentration in the nutrient solutions was monitored with an automatic DO control system (Quantum-25, Quantum Analytical Instruments Inc., USA). (3) Hypoxia + CaCl_2_ treatment: 1/2 Hoagland’s solution + 4 mM CaCl_2_ with DO of 1.0 ± 0.1 mg L^−1^. The oxygen concentration in the nutrient solutions was controlled as in the hypoxia treatment.

### Protein extraction

For analysis of total protein, root samples were harvested 3 days after the end of hypoxic treatment. Protein extraction was performed according to a modified version of the method of Hurkman [85]. Root samples (1–2 g fresh weight) were ground in a mortar with liquid nitrogen. The ground samples were suspended in 30 mM 2-amino-2-(hydroxymethyl)-1,3-propanediole (Tris)-HCl (pH 8.7) containing 1 mM ethylene glycol-bis(2-aminoethylether)-N,N,N’,N’-tetraacetic acid (EGTA), 1 mM dithiothreitol (DTT) and 1 mM phenylmethyl sulfonyl fluoride (PMSF), and then centrifuged at 15,000 g for 20 min. An aliquot (1 ml) of the resulting supernatant was placed into a tube and precipitated with acetone containing 10% TCA and 0.07% β-mercaptoethanol. The resulting protein sample was allowed to precipitate overnight at −20°C and then centrifuged at 20,000 g for 25 min. The pellet was rinsed three times with cold acetone containing 0.07% β-mercaptoethanol and allowed to stand at −20°C for 1 h. Finally, the protein pellet was air-dried and used for 2-DE.

### 2-DE

Isoelectric focusing (IEF) was performed according to the methods of Duncan and Hershey [86] and O’Farrell [87]. The dried protein pellet was rehydrated in rehydration buffer: 7 M urea, 2 M thiourea, 4% 3-[(3-cholanidopropyl) dimethylammonio]-1-propanesulfonic acid (CHAPS) (w/v), 40 mM DTT, 0.5% (v/v) immobilized pH gradient (IPG) buffer 4–7 and 0.01% (w/v) bromophenol blue. Protein levels were quantified according to the Bradford method [88]. IPG strips of nonlinear *pI* 4–7 (13 cm) were loaded with 250 μl of protein sample containing 800 μg protein in a rehydration tray for 12–16 h at room temperature. Following rehydration, the IPG strips were run on an Ettan IPGphor 3 (GE Healthcare, USA). The voltage for IEF was set at 200 V for 1 h, followed by 500 V for 1 h, 1000 V for 1 h, 3000 V for 30 min, 5000 V for 30 min, gradient 8000 V for 30 min, and 8000 V rapid focus, reaching a total of 35,000 V h. The cell temperature was maintained at 20°C with a maximum current of 50 μA per strip. After running the first dimension, IEF strips were equilibrated for 15 min with 10 ml DTT buffer containing 6 M urea, 30% (v/v) glycerol, 2% SDS, 1% (w/v) DTT and 50 mM Tris–HCl (pH8.8) and then with iodoacetamide buffer with 2.5% (w/v) iodoacetamide instead of DTT for 15 min.

The second dimensional SDS-polyacrylamide gel electrophoresis (SDS–PAGE) was carried out on running gels (Hoefer SE600 Ruby Standard Vertical System, GE Healthcare; 12.5% polyacrylamide) in the presence of SDS, as described by Laemmli [89]. The strips were embedded on the top of the SDS-gel and then sealed using a 1% molten agarose solution. Electrophoresis was carried out at 15 mA per gel until the bromophenol blue dye front reached about 1 cm from the bottom of the gel.

### Image acquisition and analysis

For Coomassie brilliant blue (CBB) R-250 staining, the gels were fixed overnight in a mixture of MeOH–H_2_O (1:1, v/v) and AcOH:H_2_O (1:9, v/v) and then stained for 2 h in a mixture of AcOH:H_2_O(1:9, v/v) and 0.1% (w/v) CBB R-250. The stained gels were destained in a mixture of MeOH-H_2_O (1:1, v/v) and AcOH: H_2_O (1:9, v/v). The CBB-stained 2-D gels were scanned using an Image scanner III (GE Healthcare). The digitized images were analyzed with Imagemaster™ 2D Platinum version 5.0 (GE Healthcare). At least three gels from each treatment in three independent experiments were used for the analysis. The intensities of spots were quantified based on their relative volume, which was determined by the ratio of the volume of a single spot to the whole set of spots. Only spots with significant (at least 1.5-fold quantitative changes) and reproducible changes in three replicates were used for mass spectrometry. Student’s *t*-test and a significance level of 95% were used for the statistical analysis of the gels. Only the spots showing a statistically significant difference in protein abundance between the treatments were considered differentially expressed spots.

### In-gel protein digestion, mass spectrometry and database search

Differentially expressed protein spots were excised from gels and transferred to sterilized 0.5 ml tubes. The excised protein spots were destained for 20 min with 100 mM NH_4_HCO_3_ in 30% acetonitrile (ACN) and then washed in Milli-Q H_2_O. The spots were kept in 0.2 M NH_4_HCO_3_ for 20 min and then lyophilized and rehydrated. Each spot was digested overnight in 30 μl of 50 mM NH_4_HCO_3_ containing 50 ng trypsin (Promega, Madison, WI, USA). After overnight digestion at 37°C, the peptides were extracted three times with a mixture of 50% ACN and 0.1% CF_3_CO_2_H (TFA). Extracts were pooled together and lyophilized. The resulting lyophilized tryptic peptides were kept at −80°C until mass spectrometric analysis.

### MALDI-TOF/TOF MS analysis and database searching

MS and MS/MS spectra were obtained using the ABI 4800 Proteomics Analyzer MALDI-TOF/TOF (Applied Biosystems, Foster City, CA, USA) operating in a result-dependent acquisition mode. Peptide mass maps were acquired in positive ion reflector mode (20 kV accelerating voltage) with 1000 laser shots per spectrum. Monoisotopic peak masses were automatically determined within the mass range 800–4000 Da, with a signal to noise ratio minimum set to 10 and a local noise window width of *m*/*z* 250. The most intense ions were selected as precursors for MS/MS acquisition, excluding common trypsin autolysis peaks and matrix ion signals. In MS/MS-positive ion mode, spectra were averaged, collision energy was 2 kV, and default calibration was set. Monoisotopic peak masses were automatically determined with a signal to noise ratio minimum set to 5. The MS, together with MS/MS spectra were searched against the NCBI viridiplantae (V.2010.12.10, 184045 sequences) and NCBI EST viridiplantae databases (V.2010.12.10, 1847412 sequence) using the software GPS Explorer™, version 3.6 (Applied Biosystems) and MASCOT version 2.1 (Matrix Science, London, UK). The parameters used for searching were: trypsin cleavage, one missed cleavage allowed; carbamidomethyl (C) set as a fixed modification; oxidation of methionines allowed as variable modification; peptide mass tolerance within 100 ppm; fragment tolerance set to ± 0.3 Da; and minimum ion score confidence interval for MS/MS data set to 95%.

### RT-PCR analysis

Total RNA was extracted from roots as described in the TRI reagent protocol (Takara Bio Inc). For all samples, total RNA (1 μg) was converted to cDNA using a Superscript first-strand synthesis system for RT-PCR according to the manufacturer’s instructions (Takara Bio Inc).

Primers were designed from the peptide sequences obtained after mass analysis according to NCBI and cucumber databases (cucumber.genomics.org.cn). Gene-specific primers used for PCR are shown in Table 3. PCR conditions were optimized for each primer set. PCR was carried out by denaturing the cDNA at 94°C for 5 min; followed by 30 cycles of 94°C for 30 s, annealing temperature (shown in Table 3) for 30 s, and extension at 72°C for 35 s. The final PCR extension step was at 72°C for 7 min. The amplified cDNA fragments were separated by 1% agarose gel electrophoresis.

**Table 3:** Primer sequences used in RT-PCR

**Transcripts**	**Product length** (***bp***)	**Annealing temp. (°C)**	**Primer pairs**
*ahd*	714	57	S 5^′^-TCAAGGTCGCCAATCCCA-3^′^
			AS 5^′^-TATGCCAGCAGCCTCAAAC-3^′^
*vatps*	427	57	S 5^′^-GGCAGTGTTACTATTGTCGG-3^′^
			AS 5^′^-TATTACGCATCATCCAGACC-3^′^
*adh*	415	57	S 5^′^-AGGGTTCATCTGTTGCTATCT-3^′^
			AS 5^′^-GGAATGTCAGTTCTCGGTTT-3^′^
*ela*	455	55	S 5^′^-GTGGATTCGCTCCTAACA-3^′^
			AS 5^′^-TTTCACAGCCTCAATACTCT-3^′^
*ss*	318	57	S 5^′^-GAGTTTGAGGTCCAGACTATTT-3^′^
			AS 5^′^-AATTTAACTGCTGCCTGATT-3^′^
*gsk*	663	53	S 5^′^-TGCCAGTTGGACTTGACG-3^′^
			AS 5^′^-GGGATGCTCGCTGATACA-3^′^
*gs*	427	55	S 5^′^-CCAGGAGAAGACAGTGAA-3^′^
			AS 5^′^-AGATGTAACGAGCAACCC-3^′^
*pdh*	437	55	S 5^′^-CCCCAAATCTACCGTCTC-3^′^
			AS 5^′^-AACTCCACAACAGGCTTC-3^′^
*mdh*	294	53	S 5^′^-TGAATGGCGTAAAGATGG-3^′^
			AS 5^′^-GGATGGAAGGAGCAAACT-3
*pod*	463	57	S 5^′^-ATTCGCCTCCATTTCCAT-3^′^
			AS 5^′^-GGCTTCCAGTTCCGTTGA-3^′^
*atpsβ*	459	57	S 5^′^-TTGACCAGGCAACGGAACA-3^′^
			AS 5^′^-TACGACCAAGCAAAGCAGACAC-3^′^
*ald*	546	57	S 5^′^-CTACAGAGGCAAATACGCT-3^′^
			AS 5^′^-TCAGGCTCCACAATAGGT-3^′^
*actin*	290	58	S 5^′^-CCGTTCTGTCCCTCTACGCTAGTG-3^′^
			AS 5^′^-GGAACTGCTCTTTGCAGTCTCGAG-3

S: Sense primer; AS: Anti-sense primer.

### Western blot analysis

The protein was extracted from roots using a mixture containing 0.5 M Tris–HCl (pH 6.8), 20% (v/v) glycerol, 2% (w/v) SDS, 5% (v/v) β-mercaptoethanol and 0.01% (w/v) bromophenol blue. The extracted protein was quantified by the Bradford method [88], denatured at 95°C for 3–5 min and then stored at −20°C until analysis.

SDS-PAGE was performed according to the method of Laemmli [89]. After electrophoresis, protein bands were visualized with Coomassie blue R250. For western blot analysis, proteins (15 μg from each sample), separated by SDS-PAGE as above, were transferred to a 0.45 μm PVDF membrane and detected with antibodies (produced in rabbit; Univ-bio, Shanghai, China) raised against ADH (AS10_685), PDC (AS10_691), and SAM (positive control). The membrane was blocked with 5% nonfat dry milk for 2 h and washed with TBST three times. The membrane was then probed with the appropriate rabbit primary antibody at a 1: 2000 dilution in TBST supplemented with 5% nonfat dry milk. After an overnight incubation at 4°C, the membrane was washed with TBST and incubated at room temperature for 1 h with a Goat Anti-Rabbit IgG HRP-conjugate (1:1000 dilution with 5% dry milk) in TBST. The membrane was then washed with TBST three times and developed using diamino benzidene (DAB) and H_2_O_2_.

## Competing interests

The authors declare that they have no competing interests.

## Authors' contribution

HLZ carried out the 2-DE experiments and mass spectrometry analysis. LXM carried out the western blot experiments. LB and YYJ participated in the RT-PCR experiment. TJ and LJ participated in sample collection and protein extraction. GSR conceived, designed, and coordinated this study. All authors read and approved the final manuscript.

## Supplementary Material

Additional file 1 **Table S1.** Effect of Ca^2+^ on biomass of cucumber seedlings under hypoxia stress [14]. Click here for file
