# Supplementary material for: Proteomic analysis of the effects of exogenous calcium on hypoxic-responsive proteins in cucumber roots
Source: Proteome Sci. 2012 Jul 12;10:42. doi: 10.1186/1477-5956-10-42 (PMC3576256; doi:10.1186/1477-5956-10-42)
Supplement: Additional file 1 — Table S1. Effect of Ca2+ on biomass of cucumber seedlings under hypoxia stress [14]. [file 1477-5956-10-42-S1.doc]

Table 1 Effect of Ca^2+^ on biomass of cucumber seedlings under hypoxia stress

| Treatment Fresh weight of Fresh weight of Dry weight of Dry weight of  shoot per plant/g root per plant/g shoot per plant/g root per plant/g | | | | |
| --- | --- | --- | --- | --- |
| CK | 10.32±0.12^c^ | 2.54±0.05 ^c^ | 0.860±0.07 ^b^ | 0.189±0.00 ^c^ |
| Hypoxia | 8.21±0.08 ^a^ | 1.71±0.02 ^a^ | 0.748±0.01 ^a^ | 0.162±0.01 ^a^ |
| Hypoxia+Ca^2+^ | 9.89±0.09 ^b^ | 2.05±0.06 ^b^ | 0.823±0.01 ^b^ | 0.182±0.01 ^b^ |

Note: 1) Different letters in each column mean significant difference at 5% level in the same time of treatment. 2) CK: Control; Hypoxia: the treatment under hypoxia stress; Hypoxia+Ca^2+^: the hypoxia treatment with CaCl_2_.
